# Australian GPs’ experiences, practices, and perspectives on postpartum care, contraception, and breastfeeding

**DOI:** 10.1093/fampra/cmaf055

**Published:** 2025-08-01

**Authors:** Keersten Cordelia Fitzgerald, Melissa Kang, Kirsten I Black

**Affiliations:** General Practice Clinical School, Faculty of Medicine and Health, The University of Sydney, Sydney, New South Wales 2050, Australia; General Practice Clinical School, Faculty of Medicine and Health, The University of Sydney, Sydney, New South Wales 2050, Australia; Obstetrics, Gynaecology and Neonatology, Central Clinical School, Faculty of Medicine and Health, The University of Sydney, Sydney, New South Wales 2050, Australia

**Keywords:** obstetrics, postpartum care, reproductive health, family planning, contraception, breastfeeding, primary care, maternity care, women’s health, gynecology

## Abstract

**Background:**

Unintended pregnancies and short interpregnancy intervals (IPIs) are common and can be associated with adverse neonatal and maternal outcomes. Effective postpartum contraception could provide women with more control over their reproductive outcomes. Lactational amenorrhoea can be effective contraception; however, early breastfeeding discontinuation is common. This study aimed to explore and understand the experiences, practices, and perspectives of Australian general practitioners (GPs) in relation to postpartum care, contraception, and breastfeeding.

**Methods:**

Twenty-one qualitative, semi-structured interviews were conducted with GPs working in Sydney, Australia. Interviews were audio-recorded and transcribed for directed content analysis and thematic analysis.

**Results:**

Directed content analysis identified a diverse range of issues that constitute postpartum care. Thematic analysis identified four themes:

(1) GPs have a holistic view of the postpartum period and play a coordinator role in postpartum care.

(2) GPs identify opportunities for empowering postpartum women in their healthcare.

(3) GPs perceive that women deprioritize their postpartum care and contraception.

(4) GPs identify barriers and facilitators for postpartum care delivery.

Subthemes provided further detail about how GPs consult with postpartum patients and opportunities to improve care. They noted areas of professional development needs and discussed the system, professional and patient factors impacting care.

**Conclusions:**

We identified several areas for improving postpartum care, including routine antenatal contraception counselling, revisiting the timing of postpartum visits, improving GP education in IPIs and breastfeeding, and improving engagement in postpartum care services through patient education.

**Key messages**

- Postpartum unintended pregnancies and short interpregnancy intervals are common.
- Postpartum contraception may give women more control over their family planning.
- Australian GPs feel women deprioritize their postpartum care and contraception.
- Australian GPs identified strategies to improve postpartum care and contraception.
- Strategies include antenatal contraception counselling and increased GP visits.
- GP and patient education may improve awareness and provision of postpartum care.

## Introduction

Unintended pregnancies and short interpregnancy intervals (IPIs) are common among postpartum women worldwide [1–3]. An IPI is defined as the period between birth of one, and conception of a following, pregnancy. The World Health Organization recommends an IPI of at least 24 months [4]. Short IPIs are associated with adverse neonatal and maternal outcomes including stillbirth, preterm labour, gestational diabetes, placental abruption, and uterine rupture [3, 5]. One Australian study found that 20.9% of women had an IPI < 12 months, and only 7.5% of these women believed this to be ideal [3]. Having young children is a common reason for the termination of pregnancy [6].

Effective contraception in the postpartum period could reduce short IPIs, providing women more control over their reproductive outcomes [1, 7–9]. There are safe, effective contraceptive methods available for postpartum women, including long-acting reversible contraception (LARC) [10]. LARCs reduce the risk of rapid repeat pregnancy compared to those using other contraception or no method [1, 7–9]. However, rates of postpartum contraception remain low and less effective short-acting methods are commonly used [11–13]. Lactational amenorrhoea can also be effective contraception for exclusively breastfeeding women up to 6 months postpartum who are not menstruating [14]. However, only 39% and 15% of infants in Australia are exclusively breastfed by 3 and 6 months postpartum, respectively [15]. Reasons for discontinuation include perceived poor milk supply, breast/nipple pain, and maternal employment [16].

Postpartum care in Australia is usually the responsibility of general practitioners (GPs). This generally includes a ‘6-week check’, a holistic check for the infant and mother, although there is growing global recognition that this time frame is not evidence-based [17–19]. There has been limited research in Australia among GPs about postpartum contraception, with only one recent paper which focused on contraception at the 6-week check [20]. The aim of this study was to explore the experiences, practices, and perspectives of Australian GPs on postpartum care, in order to identify areas for improvement.

## Methods

### Participants and recruitment

Participants were GPs practising in a diverse Health District of metropolitan Sydney, Australia. We purposively sampled to cover the geographic area and include males. Three recruitment strategies were employed: (i) emails were sent to practices of over 600 GPs who provide ‘shared antenatal care’ (model of care where antenatal care is shared between the GP and the public hospital). Emails were sent at weekly intervals up to a maximum of three times (ii) study promotion through the ‘Primary Health Network’ (PHN) newsletter (PHN is a geographically based organization which coordinates primary care) (iii) study promotion via emails and newsletters among collegiate networks at the University of Sydney.

### Data collection

All research team members, two GPs and one obstetrician-gynaecologist, contributed to the development of the interview questions. The interview was piloted with a practising GP, resulting in minor changes to improve clarity. Data were collected via in-depth semi-structured interviews with researcher K.F., on Zoom or face to face as per the GP’s preference, between April and October 2023. Prior to the interview, a brief survey captured demographic and practice data, including age, gender, languages spoken, cultural background, practice postcode, practice size, years in practice, number of sessions worked per week, and antenatal shared care status.

The interview guide explored GPs’ experiences, practices, and perspectives on postpartum care, contraception, breastfeeding, and engagement with GP services (Appendix 1). Interviews were audio-recorded and transcribed. All transcripts were de-identified and participants had the option to review their transcripts. Participants received an AUD50 book/movie voucher for their participation.

### Data analysis

Two authors (K.F., M.K.) coded five transcripts independently and discussed discrepancies, and then K.F. coded the remaining transcripts. Directed content analysis [21] was used to document the components of postpartum care. This involved quantifying how many times a component of care was raised by the participants as being included in the postpartum check, see Table 2. Thematic analysis [22] was applied to the data to identify GPs’ experiences of postpartum care, barriers and facilitators of postpartum care, and recommendations for practice. The process involved six phases of thematic analysis as per Braun and Clarke: familiarization, initial coding, searching for themes, reviewing themes, defining/naming themes, and producing the report [22]. The authors are all cis-gender females with a professional interest in women’s health. Their clinical practice and women-centred approach to care influenced their interpretation of the data, while their professional roles as either GPs (junior and senior) or obstetrician-gynaecologists influenced their consideration of the health system context. All the authors reflected on how their own experiences may influence their interpretation and worked collectively to minimize potential bias.

**Table 2.** Components of postpartum care as mentioned by 21 GP participants (2023).

Components of postpartum (total participants = 21)	*n*	%
Mental health/well-being	20	95.2
Contraception	18	85.7
Infant feeding	16	76.2
Birth history	15	71.4
Wound healing/perineal check	13	61.9
Pregnancy history/complications of pregnancy	11	52.4
Social history/support	11	52.4
Lochia	10	47.6
Cervical screening test	8	38.1
Bowel/bladder/pelvic floor	6	28.6
Breast check	4	19.0
Bonding with baby	3	14.3
Sexual issues	2	9.5
Domestic violence screen	1	4.8
Other medical issues *(included skin, blood pressure check, weight management, vaccinations, abdominal muscle separation, back pain, thrombosis screen, thyroiditis/endometritis screen, pain management, vitamin/mineral deficiencies)*	11	52.4

### Ethics

Ethics approval was granted by the University of Sydney Human Research Ethics Committee (2022/913).

## Results

### Participant demographics

**Figure 1.**
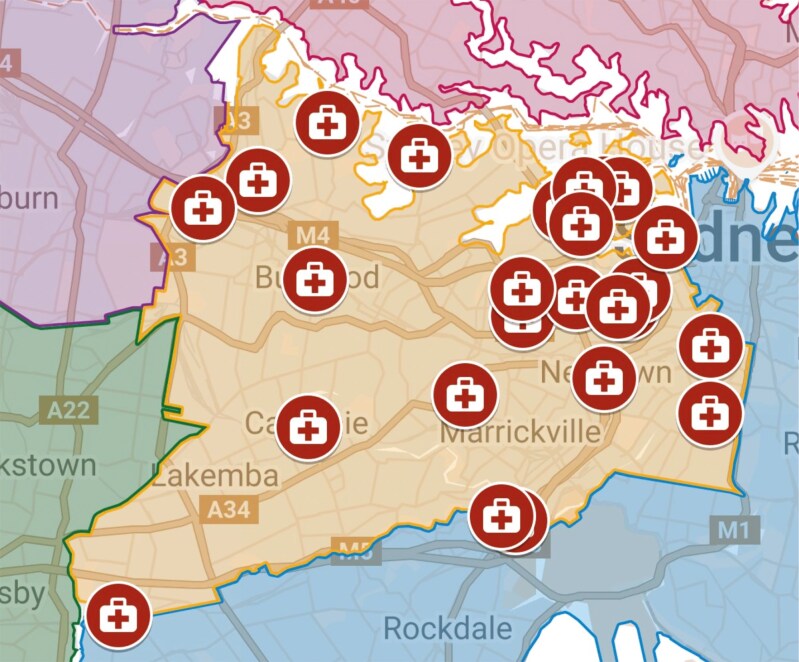
Practices worked at by the GP participants within the Sydney district by postcode. Made with google maps [23].

Twenty-one interviews were conducted with GPs. Eighteen identified as female and three as male, the age range was 27–54 years and over half spoke a language other than English. Table 1 summarizes the sociodemographic and practice characteristics of the sample. Figure 1 displays the geographic spread of practices [23]. Table 2 demonstrates the broad range of components of postpartum care identified by participants.

**Table 1.** Demographics of 21 GP participants (2023).

Demographics (total = 21)	*n*	%
Age		
* 25–34 years*	5	23.8
* 35–44 years*	12	57.1
* 45–54 years*	4	19.0
* 55 + years*	0	0.0
Gender		
* Female*	18	85.7
* Male*	3	14.3
* Other*	0	0.0
Languages spoken^a^		
* One language (English)*	10	47.6
* Two languages*	8	38.1
* Three languages*	3	14.3
Cultural background, grouped by continent^b^		
* Oceania (Australia)*	9 (8)	42.3 (38.1)
* Asia*	12	57.1
* Africa*	2	9.5
* Europe*	4	19.0
* North America*	0	0.0
* South America*	0	0.0
Size of practice^c^ (total number of practices worked at by the participants in the SLHD = 25)		
* Solo practice (1 GP)*	0	0.0
* 2–5 GPs*	9	42.9
* 6–10 GPs*	12	57.1
* 11 + GPs*	4	19.0
Years in general practice		
* 0–5 years*	4	19.0
* 6–10 years*	9	42.9
* 11–20 years*	7	33.3
* > 20 years*	1	4.8
Number of sessions per week worked in general practice^d^		
* 1–2 sessions*	1	4.8
* 3–4 sessions*	4	19.0
* 5–6 sessions*	6	28.6
* 7 + sessions*	10	47.6
Registered as an antenatal shared care provider in the SLHD		
* Yes*	19	90.5
* No*	2	9.5
Number of postpartum patients per week on average		
* < 1*	4	19.0
* 1–4*	10	47.6
* 5–9*	4	19.0
* > 10*	3	14.3

^a^Languages other than English include: Arabic, Cantonese, Greek, Hindi, Indonesian, Mandarin, Russian, Shanghainese, Spanish, Tamil, Thai, and Vietnamese.

^b^Some participants identified with multiple cultural backgrounds.

^c^Some participants work at multiple practices within the SLHD.

^d^This includes sessions that participants may work at practices outside the SLHD.

Four themes were identified:

1. GPs have a holistic view of the postpartum period and play a coordinator role in postpartum care;
2. GPs identify opportunities for empowering postpartum women in their healthcare;
3. GPs perceive that women deprioritize their postpartum care and contraception; and
4. GPs identify barriers and facilitators for postpartum care delivery.

### Theme 1—GPs have a holistic view of the postpartum period and play a coordinator role in postpartum care

#### How GPs define the postpartum period

Participants defined the postpartum period as extending beyond the traditional 6 weeks; with definitions such as until breastfeeding cessation or up to 2 years, particularly if there were postpartum mental health concerns:

*typically, it’s up to six weeks. But the issues doesn’t just end there, it can go on…even to one year I still see some postpartum issues* (GP2)

All participants identified 6 weeks as the usual time for the postpartum check, however reported that women often present for support earlier. They expressed concern that by waiting until 6 weeks, opportunities for early intervention are missed, especially breastfeeding issues, which may contribute to earlier discontinuation:

*ideally sort of in the first couple of weeks. Cos then if anything, like you sort of address things before they become a big problem* (GP15)

#### Holistic approach to postpartum care

Participants viewed their role in postpartum care as holistic and broad:

*physical wellbeing of the woman, how they’re going as a new mum, supporting them mentally through that and the changes associated with that. So… a broad spectrum… physical and mental health* (GP7)

#### Coordination role

Participants felt they were the first port of call and had a responsibility to coordinate care:

*as a GP, that’s our role to kind of coordinate all this care, to organise and coordinate this care* (GP14)

Participants recommended a multi-disciplinary team either as required, tailored to the patient’s needs, or routinely, using a team-based approach with shared responsibility with other professionals such as community nurses and midwives. Participants valued input from other health professionals including mental health services, lactation consultants, maternal child health nurses, pelvic floor physiotherapists, midwives, dietitians, obstetricians, and other specialists. Participants also referenced the benefit of midwife home visits, stating that GPs are not set up for this service.

### Theme 2—GPs identify opportunities for empowering postpartum women in their healthcare

#### Encouraging engagement with postpartum services

Participants reported variable engagement of women in their postpartum care, from very engaged to those who were difficult to follow-up, necessitating opportunistic healthcare. Participants felt some women were not aware of the 6-week check, particularly those without a regular GP or private obstetrician; others were aware of this recommendation but did not book in. Women were often unfamiliar with the components and importance of a 6-week check which contrasted with their awareness of their baby’s check. Participants felt that more education for women is needed, including antenatally:

*when I or other people do mention about what needs to be done in terms of their postpartum care then they become engaged because they know that it’s important* (GP2)

Suggestions included integrating maternal and newborn resources on discharge, including timelines for health checks. Participants felt that it would be beneficial for other health professionals to remind women of their postpartum checks.

#### Facilitating contraception

Participants were confident discussing contraception with postpartum patients, offering the full range of options. Many thought discussing contraception antenatally could improve postpartum contraception, but rarely did so due to time and resource barriers. Participants believed that contraceptive choice needed to be individualized:

*The best contraception is … the one the mother uses* (GP16)

However, some felt that IUDs were underutilized overall.

Factors that influenced participant’s recommendations included medical contraindications, patient preference, family planning, and breastfeeding. For example, participants felt that a woman may not ‘want the hassle’ (GP16) of having an IUD inserted if they wish to have another child soon. Other frequently considered factors included the patient’s prior contraception, side effects, and ease of adherence.

Participants felt that lactational amenorrhoea was not a reliable method of contraception and either explained its limitations to women to allow for shared decision-making or recommended other options:

*in my mind it’s highly unreliable and it’s not really a method of contraception* (GP17)

Participants reported variable levels of patient knowledge around contraception. Commonly participants felt that their patients were not familiar with the breadth of contraceptive options and often viewed oral options as the ‘default’ (GP4). Participants felt that women often wanted to return to the contraception they used previously. Participants described barrier methods and oral contraceptives (particularly progesterone-only pills) as the most commonly used, with a small uptake of IUDs. Participants felt that they had a responsibility to educate women about their contraception options and the risk of unplanned pregnancy, to allow women to make informed choices:

*We’d like to give you [patients] control over when you’d like to have another baby* (GP16)

Participants stated that partners would rarely be involved in contraception discussions unless a vasectomy was being considered. Even then, one participant pointed out that it is often the women’s responsibility to raise this with their partner and send them to the GP:

*I’ll suggest it as a method [vasectomy] and we’ll talk about the efficacy and the and the pros and cons and then if that’s something they’re interested in, then they would send the husband in to discuss it directly with the doctor* (GP17)

### Theme 3—GPs perceive that women deprioritize their postpartum care and contraception

#### Postpartum care

Participants felt that women prioritized their baby’s healthcare over their owns and that some women, particularly multiparous women or women without active concerns, did not recognize the importance of postpartum care. Participants described techniques to address this, such as always booking the mother an appointment at the same time as the baby’s appointment, providing ‘opportunistic’ (GP4) maternal care during the infant’s appointment and using the baby’s care to engage women:

*I often do say to patients come at two to four weeks … I put the baby on the scales and use that as an excuse* (GP18)

However, it was acknowledged that having the baby present in the consultation could be a distraction.

#### Contraception

Participants reported that some women were interested in contraception, whereas others would ‘laugh’ (GP12, GP13, GP18, GP21), ‘giggle’ (GP), or be ‘surprised’ (GP2, GP15) that it was being brought up. Participants felt that women need time to make contraceptive choices in this period and often delay decision-making. Therefore, contraception provision frequently occurred over multiple appointments and some women were lost to follow-up. Access to LARCs in particular was delayed due to the need for additional appointments, so less effective interim methods such as condoms or oral contraception were used.

It appeared that contraception was not commonly a priority as many postpartum women were not yet sexually active and had competing interests:

*Sometimes they just kind of laugh and say, you know, there’s nothing happening, because life’s so busy with the new baby or they’re not ready* (GP21)

Despite this, participants felt that women were open to discussing contraception, although many declined to commence.

### Theme 4—GPs identify barriers and facilitators for postpartum care delivery

#### System factors

Antenatal shared care and continuity of care were raised by participants as a facilitator of postpartum care. Participants felt that a strong doctor–patient relationship throughout pregnancy and postpartum could improve 6-week check booking/attendance and contraception uptake:

*if it’s [a script for contraception] from the obstetrician, they’ve got a trust relationship there already, but sometimes from the hospital it will have been a junior doctor or someone they don’t know, so then they want to talk to me about it first* (GP21)

Participants identified that although the cost of contraception was not usually a significant concern for their patients, it may limit their options, in particular IUDs and drosperinone 4 mg (a more recently available progesterone-only pill). In contrast, in practice access to IUDs was seen to be a facilitator for their uptake:

*when we say, oh and you can get that done here [IUD insertion], it’s like, oh well I’ll just get that…whereas if you say you’ve got a waitlist and family planning will do it, you know, sometime in the next… then it’s like, oh it’s too hard* (GP17)

Nearly all participants described seeing minimal immediate postpartum contraception in their patients, with only occasional inpatient IUD or etonogestrel subdermal implant insertion, or sterilization during Caesarean section. Some participants believed that immediate postpartum contraception would be convenient for women:

*one of my wishes … is that the LARCs are offered immediately after birth. I know that they can be put in immediately … because it is a lot harder [in the community], because women are just busy after they give birth to kind of go okay well, you need a 45 minute, like, you know, pre insertion consultation and then you need to come back on another day for me to insert it* (GP5)

Participants had varied confidence in managing breastfeeding consults, with some managing complex cases and others referring to lactational consultants or other GPs. Participants felt that they had very limited education about breastfeeding, although some had sought out additional courses to upskill:

*in GP training, we don’t really get taught very much about [breastfeeding] but oftentimes we are the first port of call where people come and see us* (GP10)

Participants reported they would value further breastfeeding education. Participants who had personal experience with breastfeeding reported feeling significantly more confident managing breastfeeding consultations after breastfeeding themselves:

*I’m a lot more confident now having gone through [breastfeeding] myself* (GP8)

Participants identified that after the 6-week check, there is no formal follow-up for women. Therefore, if women delay any decisions, such as contraception, they may be lost to follow-up:

*they hardly ever come back. And you know there’s no other formal follow-up for a mum really, in the postpartum period… once they do that six week check it’s almost like, you know that’s it* (GP1)

Participants reported that lack of practitioner time was a barrier to providing thorough postpartum care. Participants believed an extended appointment was required for a postpartum check, however, inadequate financial incentive for this type of care was raised as a barrier:

*It’s not well funded to provide the comprehensive support* (GP21)

Interestingly, despite being aware of the current lack of postpartum care guidelines, participants felt that guidelines for standard care were not routinely required. Some participants reported using other guidelines for the various components of postpartum care if there were specific issues, such as guidelines for mastitis:

*things like infections that you’re concerned about, there’s the therapeutic guideline, endometritis and mastitis… I would say a lot of it [postpartum care], yeah, maybe the bulk of it … I don’t use any guidelines for* (GP3)

It was also highlighted that general postpartum care guidelines may be helpful for GP trainees or GPs with limited women’s health exposure:

*Because I don’t do it so much anymore, I always look it up pretty much any antenatal, postnatal care* (GP17)

#### GP factors

One of the male participants felt that he had minimal clinical exposure to postpartum patients due to his gender, despite being interested and having experience in obstetrics:

*not a lot of women wanted to come to the one male GP in the practice* (GP13)

Two male participants also reported difficulty in maintaining knowledge and skills without ongoing clinical exposure:

*you immediately deskill in all the other stuff* (GP13)

Participants did not feel confident regarding recommended birth intervals, with a wide variety of time intervals suggested, and did not routinely discuss birth spacing with patients. A few participants thought birth spacing intervals should be focused on the woman’s social, psychological, and physical well-being and family planning goals, rather than a medically recommended time frame. Participants generally perceived birth spacing to be more relevant after a caesarean section. Some felt it was the obstetrician who should discuss this:

*I’ve got no idea, it’s not my job to decide* (GP4)

#### Patient factors

Participants felt that patient concern for side effects was a significant barrier to contraception, common concerns were the impact on breastfeeding, irregular bleeding, weight gain, skin effects, and mood. Some of these concerns were reported to be from the patient’s prior experiences or from family and friend experiences:

*my girlfriend had one and loved it, or my friend had one and hated it, you know it all impacts* (GP11)

Two participants also referred to the growing wellness movement and patients’ desires to avoid hormonal contraception.

Participants frequently raised the patient’s cultural background as an influencing factor in contraceptive choice. Notably, participants were from a wide range of cultural backgrounds themselves. Participants felt that some cultural groups may be more or less likely to initiate contraception discussions or uptake certain contraception:

*95% of [one ethnic] background women will choose condom rather than anything else. But the other demographic would choose anything other than condoms* (GP2)

*occasionally someone of certain faiths might not like the idea of contraception as in they don’t believe in it* (GP4)

Lack of support for postpartum women was commonly described as a barrier to postpartum care, contraception, and breastfeeding continuation. For example, women without a support system, particularly for childcare, may be limited in their ability to attend appointments such as for an IUD insertion.

*if women have family members, partners, mums, whatever who’s who are available I find it’s easier to encourage them to come in for that because they’re not feeling their one handed and having to juggle everything* (GP20)

Interestingly, age was raised as a potential barrier for older mothers considering contraception/birth spacing, given the issue of balancing this with fertility:

*a lot of older mothers they probably do know [about birth spacing intervals] but then they’re balancing it with their like “What if I can’t get pregnant again if I wait another two years” and so they’re … willing to accept that risk* (GP6)

Three participants also felt that some patients receive pressure from their partners to avoid contraception:

*Some people say it’s pressure from their partner, that their partner doesn’t want them to be on hormonal contraception while they’re breastfeeding* (GP21)

Participants felt that women were aware of the benefits of, and prioritized, breastfeeding. However poor milk supply, complications of breastfeeding, poor support, and return to work contributed to discontinuation. Participants described the importance of a non-judgmental approach to infant feeding as many of their patients experienced pressure to breastfeed and felt like a ‘failure’ (GP20) if they were unable to, which could negatively impact their well-being.

## Discussion

This study explored GPs’ perspectives on postpartum care, contraception, and breastfeeding and highlighted areas for actionable change in postpartum care provision.

Firstly, participants reported that women need time to make contraceptive decisions, and therefore often delay commencing contraception. Worldwide there are low rates of effective postpartum contraception [2, 12, 13]. Furthermore, immediate postpartum LARC insertion is very low in Australia based on limited data, despite its effectiveness [24], and demonstrated patient satisfaction [25]. Immediate postpartum insertion may facilitate LARC uptake, as participants frequently reported that women viewed the additional appointments required for LARCs as a barrier, in keeping with international literature [26–28]. Antenatal discussion may allow women more time to consider their contraceptive choices and facilitate timely uptake, including LARCs [5, 17]. Evidence suggests that women want more detailed contraception counselling both antenatally and postnatally [29–31]. Implementation of contraception counselling in routine antenatal care could improve contraception uptake among postpartum women [32, 33].

Secondly, the timing of postpartum visits needs to be reconsidered; such as a check before 6 weeks and ongoing, personalized care after 6 weeks. The literature supports the uncertainty around the timing of postpartum visits [19], with prior research demonstrating inconsistent recommendations from GPs and confusion among postpartum women [34, 35]. Given most participants reported they did not require postpartum care guidelines, more research into this issue and how to implement the timing of postpartum visits is needed. In particular, investigating the value of increased general practice input earlier in the postpartum period and recommending routine follow-up for all women at the time of their baby’s 4-month check. We postulate that these additional reviews may provide an opportunity for early intervention, preventative health, and follow-up, although as described above, there is little evidence for the timing of postpartum visits [19].

Lack of training was highlighted by participants (see theme four-‘system factors’) as a barrier to providing breastfeeding care, consistent with other research [36–38]. Since professional breastfeeding support for women improves rates of breastfeeding [39], breastfeeding education in GP training and ongoing professional development is crucial to improve GPs skills. Although, as described above, given participants were not engaged with postpartum care guidelines, it does pose the question of how to effectively disseminate this knowledge.

Participants offered suggestions to improve the engagement of women in postpartum care, such as antenatal patient education about the importance of postpartum care, which is consistent with another recent study [20]. This was also reflected in an Australian study that found women were unsure why they needed to see the GP postpartum [34]. Other suggestions to improve engagement included integrating maternal and infant resources and reminders about postpartum checks from other healthcare providers. Continuity of care was also referenced by participants as a facilitator to engagement which may be related to the opportunities for patient education, as well as trust and rapport building.

Lastly, our study found that participants did not routinely discuss birth spacing with patients and lacked confidence in IPI recommendations. Another study of GPs had similar results, with GPs lacking awareness of interconception care [40]. Another study found that less than half of women reported receiving information about IPIs [3]. Currently, it appears that education for GPs around IPIs is limited, and this may put women at risk of short IPIs without recognizing the associated risks [3, 5]. Given GPs’ limited understanding of interconception care [40], addressing GP education may be an effective strategy to improve awareness of safe IPIs.

### Strengths and limitations

This study’s qualitative design allowed for an in-depth exploration of GPs’ views, generating rich, clinically relevant data. Limitations include the skewed, self-selected sample, most of whom were antenatal shared care providers and likely to be more confident in women’s health. The high number of female participants may reflect that female GPs are more likely to provide postpartum care compared to their male counterparts. Our study also focused on GPs working in one geographic district of Sydney. These factors may affect the generalizability of these results to the broader GP community. Despite these limitations, the insights from these experienced GPs (most with a special interest in postpartum care) are relevant for all general practice and the recommendations can be widely applied beyond this local area.

## Conclusion

GPs’ holistic and patient-centred approach and their interest in supporting women’s agency is a testament to the important role that GPs play in postpartum care. We identified several areas that could enhance GPs’ postpartum care such as routine antenatal contraception counselling, revisiting timing of postpartum visits, improving GP education in IPIs and breastfeeding, and patient education to improve engagement in services.

## Data Availability

The data in this study cannot be shared publicly to maintain participant privacy but may be shared upon reasonable request to the corresponding author.

## Appendix 1: Interview guide

### Australian general practitioners’ experiences, practices, and perspectives on postpartum care: contraception and breastfeeding

#### Interview schedule

##### Demographic and practice details

- **Participant ID number:** (this will match/ be re-identifiable on the consent form)
- **Age range:**
  ◦ 25–34 years
  ◦ 35–44 years
  ◦ 45–54 years
  ◦ 55–64 years
  ◦ 65–74 years
  ◦75 + years
- **Gender**
  ◦ Female
  ◦ Male
  ◦ Other
- **Language/s spoken**
- **Cultural background/s**
- **Postcode of practice** _ _ _ _
- **Size of practice**
  ◦ Solo practice (1 GP)
  ◦ 2–5 GPs
  ◦ 6–10 GPs
  ◦ 11 + GPs
- **Years in general practice**
  ◦ 0–5
  ◦ 6–10
  ◦ 11–20
  ◦ 20+
- Number of sessions per week worked in general practice (in this practice/ area)
  ◦ 1–2
  ◦ 3–4
  ◦ 5–6
  ◦ 7+
- **Antenatal shared care registered in Sydney Local Health District**
  ◦ Yes
  ◦ No

#### Interview schedule

Date:

Interviewer:

In person/Online:

Start Time:

End Time:

#### Postpartum care: definition, guidelines, and timing

- How many postpartum women do you see on average per week? (regardless of whether they attend/you deliver postpartum care)
- How would you define or describe ‘postpartum care?’
  ◦ *(Probes: What duration after birth? What are the key elements of postpartum care?)*
- Thinking about your previous response, what aspects of postpartum care fall into the GP’s role? Following on from that, are there other aspects of postpartum care which are delivered/ can or should be delivered by other professionals (e.g. early childhood nurse, home midwife, etc.)?
- What is your experience of using guidelines for providing postpartum care?
  ◦ *(Probes: Which do you use, if any? Which are you aware of?)*
- What do you believe should be routinely involved in a postpartum check and when should these checks occur?

Postpartum care: contraception

- Can you describe whether and how you approach contraception when consulting with postpartum patients?
  ◦ *(Probes: Do you routinely discuss contraception at the postpartum visit and if so, what options do you commonly provide women? What factors influence your recommendations? When do you advise women to commence contraception? Is this something you discuss antenatally?)*
- Can you describe patients’ responses to discussions about contraception?
  ◦ *(Probe: Do you find there is high uptake of contraception when offered?)*
- Do you routinely discuss birth spacing/intervals? Does this change if it is a vaginal delivery or Caesarean section?
  ◦ *(Probes: What do you think is an* id*eal birth interval? Are patients generally aware of* id*eal birth intervals?)*
- What are the barriers and facilitators you find in the provision of contraception to this population?

Postpartum care: breastfeeding

- Can you describe whether and how you discuss breastfeeding at the postpartum visit?
  ◦ (*Probe: If the woman is breastfeeding, do you discuss lactational amenorrhoea as a method of contraception?*)
- How confident do you feel in discussing breastfeeding with your postpartum patients?
  ◦ *(Probe: What would make you more confident?)*

Postpartum care: engagement and improvements

- Looking more generally at postpartum care, can you describe the level of engagement with postpartum patients about their postpartum care?
  ◦ *(Probe/guide: Relate back to the GP’s definition of ‘postpartum care’—are they able to engage patients in all aspects of this care, are they limited by time or patient priorities—for example, women not tending to their own postpartum care but to their baby’s care)*
- What would you say are general barriers and facilitators in providing postpartum care?
- How do you think we can strengthen engagement with postpartum women to provide appropriate care?

### Conclusion

- Is there anything else you would like to share?
